# BSA-Assisted Synthesis of Au Nanoclusters/MnO_2_ Nanosheets for Fluorescence “Switch-On” Detection of Alkaline Phosphatase

**DOI:** 10.3390/bios15010049

**Published:** 2025-01-15

**Authors:** Yijiong Xue, Chengqi Bao, Hui Liu, Fanghui Ma, Minghui Yang, Xiaoqing Li

**Affiliations:** 1 Hunan Provincial Key Laboratory of Micro & Nano Materials Interface Science, College of Chemistry and Chemical Engineering, Central South University, Changsha 410083, China; xueyijiong1122@163.com (Y.X.); baochengqi0616@163.com (C.B.); lighting0201@163.com (H.L.); 202301013@csu.edu.cn (F.M.); 2 Xia Duopu Health Center of Ningxiang, Changsha 410605, China; 3 Furong Labratory, Changsha 410083, China; 4 The Department of Dermatology, Xiangya Hospital, Central South University, Changsha 410008, China

**Keywords:** Au nanoclusters, MnO_2_ nanosheets, alkaline phosphatase, fluorescence resonance energy transfer

## Abstract

A fluorescence probe for “switch-on” detection of alkaline phosphatase (ALP) was developed based on Au nanoclusters anchored MnO_2_ nanosheets (Au NCs-MnO_2_ NSs), which were synthesized using bovine serum albumin (BSA) as template through a simple one-pot approach. In the sensing system, MnO_2_ NSs function as both energy acceptors and target identifiers, effectively quenches the fluorescence of Au NCs via fluorescence resonance energy transfer (FRET). The presence of ALP catalyzes the hydrolysis of L-ascorbic acid-2-phosphate (AAP) to ascorbic acid (AA), reducing MnO_2_ NSs to Mn^2+^ and facilitate the fluorescence recovery of Au NCs. The fluorescence assay offers the advantages of facile preparation, cost-effectiveness, good specificity, and high sensitivity. Moreover, the assay exhibits a broad linear range (0.005 U/mL to 8 U/mL) for ALP detection with a remarkable limit of detection of 0.0015 U/mL. Notably, this assay demonstrates promising applicability for detection ALP in human serum samples, thereby providing valuable potential for clinical applications.

## 1. Introduction

Optical sensors have gained widespread focus in the field of sensing [1,2,3]. Especially, fluorescence technology is increasingly being utilized to detect biomolecules, ions, pesticides, and other chemicals owing to their advantages such as high sensitivity, high throughput and cost effectiveness [4]. It is worth emphasizing that selecting fluorescent probes is critical for ensuring sensor performances. Different fluorescent nanomaterials encompass organic dyes, quantum dots (QDs) [5,6,7], metal nanoclusters [8,9], upconversion nanoparticles (UCNPs) [10,11], graphitic-C_3_N_4_ [12,13] and so on have been applied in fluorescence sensing. Metal nanoclusters, in particular, have drawn great scientific attention due to their size being comparable to the Fermi wavelength of conducting electrons. Consequently, their optical and electrical properties exhibit marked differences compared to individual atoms and larger nanoparticles. The quantum confinement effect endows nanoclusters with the ability to emit intense fluorescence, and the emission wavelength can be tuned from ultraviolet to near-infrared by varying the number of constituent atoms [14,15]. Among different metal nanoclusters, copper nanoclusters (Cu NCs) have garnered significant research interest due to their facile synthesis process and cost-effectiveness. However, the susceptibility of nanoscale-Cu to oxidation in solution or air, as well as the tendency of Cu NCs to aggregate, renders its fluorescence intensity vulnerable to external environmental factors [16,17]. By comparison, Au nanoclusters (Au NCs) feature better stability and fluorescence properties, making them ideal for a wider range of applications.

Fluorescence resonance energy transfer (FRET) is a distance-dependent spectroscopic technique wherein the emission spectrum of the donor significantly overlaps with the absorption spectrum of the acceptor [18,19]. Typically, the fluorescence emitted by the fluorescent donor can be absorbed by the fluorescent acceptor, thereby leading to the occurrence of fluorescence quenching. Owing to their large specific surface area, wide absorption spectrum (250–550 nm), good biocompatibility and stability, two-dimensional (2D) MnO_2_ nanosheets (NSs) have been widely employed as energy acceptors and fluorescence quenchers for the construction of FRET sensing platforms [20,21]. In addition, MnO_2_ is easily reduced to Mn^2+^ by reducing substances, such as glutathione (GSH), cysteine (Cys), thiocholine, ascorbic acid (AA), H_2_O_2_, etc. [22,23,24]. This characteristic renders it highly suitable for fluorescence sensing. For example, Xu et al. developed a MnO_2_ in-situ coated upconversion nanosystem for determining of hypoxanthine in aquatic products [25]. Guo et al. fabricated Cys-MnO_2_ nanospheres and MnO_2_ NSs through a facile ultrasonic process to construct a FRET-based fluorescence probe for detection of GSH in human serum samples [26]. Yan et al. also synthesized a branched poly (ethylenimine) carbon dots and MnO_2_ NSs by a simple low-temperature process for determination of malachite green [27].

**Scheme 1:**
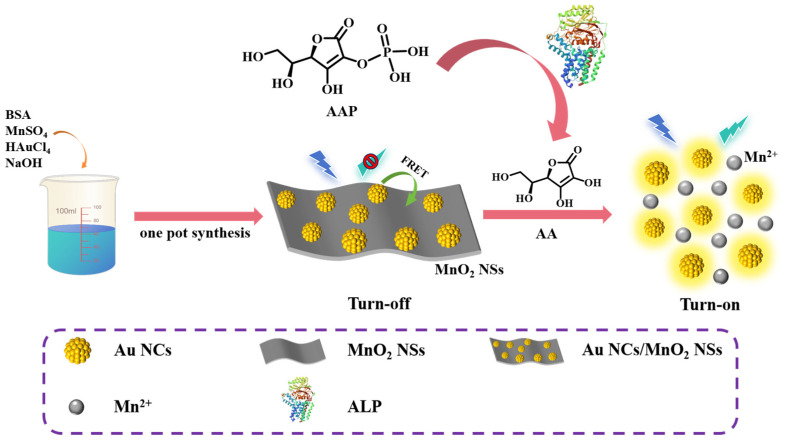
Schematic diagram of the synthesis and the principle of the FRET-based assay for ALP.

Inspired by these advancements, we employed bovine serum albumin (BSA) as template to successfully synthesize Au NCs-MnO_2_ NSs through a facile one-step approach and developed a FRET-based fluorescent probe to determine alkaline phosphatase (ALP) activity (Scheme 1). In the proposed sensing strategy, Au NCs function as the energy donor, while MnO_2_ NSs serve as the energy acceptor to achieve FRET, leading to efficient quenching of the fluorescence emitted by Au NCs. Moreover, MnO_2_ NSs also act as target identifiers for ALP recognition. The hydrolysis of L-ascorbic acid-2-phosphate (AAP) catalyzed by ALP generates AA, which can subsequently reduce MnO_2_ to Mn^2+^, thereby restoring the fluorescence of Au NCs and allowing for sensitive ALP detection. Furthermore, successful application for detection of ALP in human serum demonstrates the immense potential of the assay for clinical applications.

## 2. Experimental Section

### 2.1. Chemicals and Materials

Manganese sulfate monohydrate (MnSO_4_·H_2_O), BSA, chloroauric acid (HAuCl_4_), GSH, uric acid (UA), dopamine (DA), L-lactic acid (L-LA), protein kinase (PKA), sodium hydroxide (NaOH), and AAP were bought from Aladdin Biochemical Technology Co., Ltd. (Shanghai, China). ALP (2000 U·mL^−1^) and AA were purchased from Sigma-Aldrich (Shanghai, China). All chemicals are of analytical purity, and all solutions are prepared with ultrapure water (18.2 MΩ cm resistivity at 25 °C, Milli-Q).

### 2.2. Instruments

The transmission electron microscopy (TEM) images and energy-dispersive spectroscopy (EDS) were conducted on JEM-2100 F electron microscopy (JEOL, Tokyo, Japan). X-ray diffraction (XRD) was measured by X-ray diffractometer (XRD-7000, Shimadzu, Kyoto, Japan). X-ray photoelectron spectroscopy (XPS) was obtained by K-Alpha spectrometer (Thermo Scientific, MA, USA). The UV-vis absorption spectra were performed by UV-2450 spectrophotometer (Shimadzu, Kyoto, Japan). The fluorescence spectra were recorded on a F-7000 spectrofluorometer (Hitachi, Tokyo, Japan) with a Xe lamp.

### 2.3. Synthesis of MnO_2_ NSs and Au NCs

MnO_2_ NSs were prepared according to a previously reported method [28]. Briefly, a total of 25 mg of BSA was accurately weighed and dissolved in 550 μL of H_2_O, followed by the addition of 200 μL of MnSO_4_ solution (50 mM). Subsequently, 50 μL of NaOH solution (5 M) was introduced into the above mixture, which was then stirred in dark at room temperature for 12 h. The obtained product was then centrifugated (12,000 rpm, 10 min) and washed with water for three times. Finally, the prepared MnO_2_ NSs was dispersed in 1 mL of H_2_O and stored in the dark at 4 °C before use. The preparation method of Au NCs is similar, except that MnSO_4_ is replaced with HAuCl_4_ solution (25 mM).

### 2.4. Preparation of Au NCs-MnO_2_ NSs Probe

The Au NCs-MnO_2_ NSs composite materials were synthesized by a one pot method. Typically, BSA (25 mg) was dissolved in 550 μL of H_2_O. Then, 200 μL of MnSO_4_ solution (10 mM, 20 mM, 50 mM, 80 mM, 100 mM) and 200 μL of HAuCl_4_ (25 mM) were added to the above solution. Afterwards, 50 μL of NaOH solution (5 M) was added and stir in the dark at room temperature for 12 h. The mixture was then centrifugated (12,000 rpm, 10 min) and washed with water for three times. Finally, the prepared Au NCs-MnO_2_ NSs was dispersed in 1 mL of H_2_O and stored in the dark at 4 °C until use.

### 2.5. Detection of ALP

Typically, a series of ALP solutions (10 μL) with varying concentrations (0.005 U/mL, 0.05 U/mL, 0.5 U/mL, 1 U/mL, 3 U/mL, 5 U/mL, 6.5 U/mL, 8 U/mL, and 10 U/mL) were prepared. After that, 100 μL of AAP solution (20 mM) was added to each ALP solution at 37 °C and allow to react for 15 min. Finally, 10 μL of Au NCs-MnO_2_ complex solution was added and the reaction was carried out at 37 °C for 5 min before fluorescence testing. (Excitation: 520 nm).

### 2.6. Detection of ALP in Serum Sample

Fresh human serum sample of healthy people was obtained from the Xiangya hospital (Changsha, China). Typically, human serum samples spiked with different concentration of ALP (0.5, 1, 5 U/mL) was prepared and mixed with 100 μL of AAP solution (20 mM) for 15 min at 37 °C. Subsequently, the prepared Au NCs-MnO_2_ solution was mixed with the above mixture for 5 min and subjected to fluorescence measurement.

## 3. Results and Discussion

### 3.1. Characterization of the Au NCs-MnO_2_ NSs

**Figure 1:**
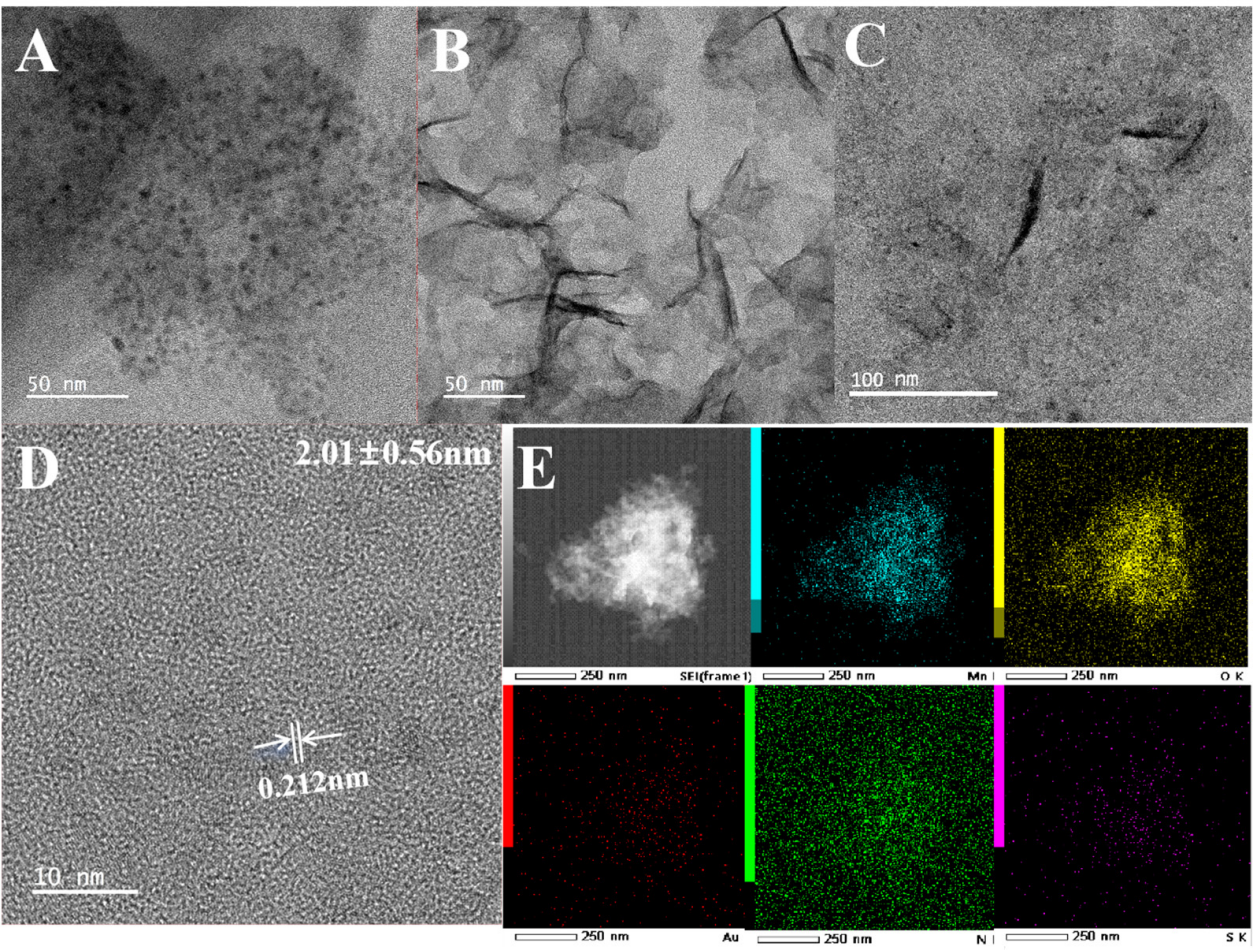
TEM image of (**A**) Au NCs, (**B**) MnO_2_ NSs and (**C**) Au NCs-MnO_2_ NSs; (**D**) HRTEM image of Au NCs-MnO_2_ NSs; (**E**) EDS element mapping photographs of Mn, Au, N, O, and S of Au NCs-MnO_2_ NSs.

Au NCs, MnO_2_ NSs and Au NCs-MnO_2_ NSs were prepared via a simple one-pot method [29]. Morphology and particle size of the three nanomaterials are observed by TEM. As show in Figure 1A and Figure S1, TEM images show that Au NCs are monodispersed and spherically shaped, with a size distribution ranging from 2–5 nm. The TEM images in Figure 1B reveal a distinct two-dimensional sheet-like structure with evident folds for the synthesized MnO_2_ NSs, which is consistent with the literature [30]. TEM was further employed to characterize the microstructure of Au NCs-MnO_2_ NSs. Similarly, TEM analysis confirmed that the Au NCs-MnO_2_ NSs possess a characteristic two-dimensional nanoplate structure, with dimensions ranging from 200 to 250 nm (Figure 1C). Moreover, high-resolution TEM (HRTEM) imaging revealed successful dispersion of Au NCs on the surface of MnO_2_ NSs, exhibiting an average size of 2.01 ± 0.56 nm and clear lattice fringes with a spacing of 0.212 nm, which is consistent with (1,1,−2) plane of MnO_2_ (Figure 1D) [29]. To further demonstrate the coexistence of Au NCs and MnO_2_ NSs, the composite was tested using energy-dispersive spectroscopy (EDS) mapping (Figure 1E), which confirmed the uniform distribution of Mn, Au, N, O, and S elements.

**Figure 2:**
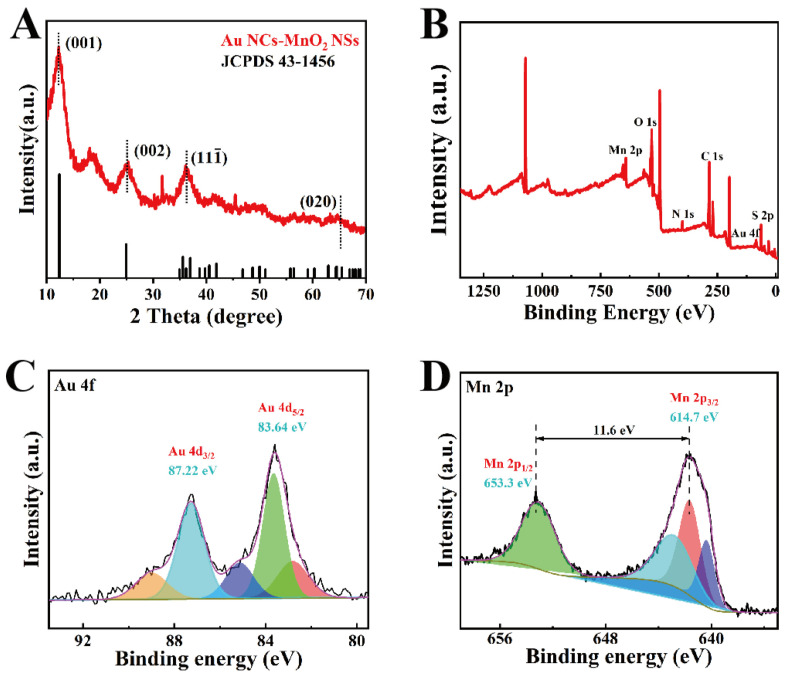
(**A**) XRD pattern of Au NCs-MnO_2_ NSs; (**B**) XPS pattern of Au NCs-MnO_2_ NSs; (**C**,**D**) The element scans patterns for Au 4d and Mn 2p of Au NCs-MnO_2_ NSs.

XRD was used for further investigation of the composition of the crystal structures. As seen in Figure 2A, the characteristic diffraction peaks of Au NCs-MnO_2_ NSs were at 12.5°, 25.2° and 37°(*2θ*), which correspond to the (001), (002), and (1,1,−1) diffraction crystal planes of birnessite-type MnO_2_ NSs crystal, respectively (JCPDS 43-1456). X-ray photoelectron spectroscopy (XPS) was applied to confirm the chemical state of element. The full scan spectrum (Figure 2B) reveals the coexistence of Mn, Au, N, O, C and S elements in the Au NCs-MnO_2_ NSs. XPS analysis of Au NCs exhibits two distinctive peaks at 87.22 eV and 83.64 eV, corresponding to 4d_3/2_ and 4d_5/2_ spin-orbit peaks of Au NCs (Figure 2C). For MnO_2_ NSs, two well-resolved peaks located at 641.7 eV and 653.3 eV were attributed to Mn (Ⅳ) 2p_1/2_ and Mn (Ⅳ) 2p_3/2_ spin-orbit peaks, respectively (Figure 2D). In addition, the observed spin-energy separation of these peaks supports earlier study findings, showing an obvious presence of Mn (Ⅳ) in the synthesized product. These results presented collectively support the effective synthesis of Au NCs-MnO_2_ NSs.

### 3.2. Feasibility Analysis of Au NCs-MnO_2_ NSs for ALP Detection

**Figure 3:**
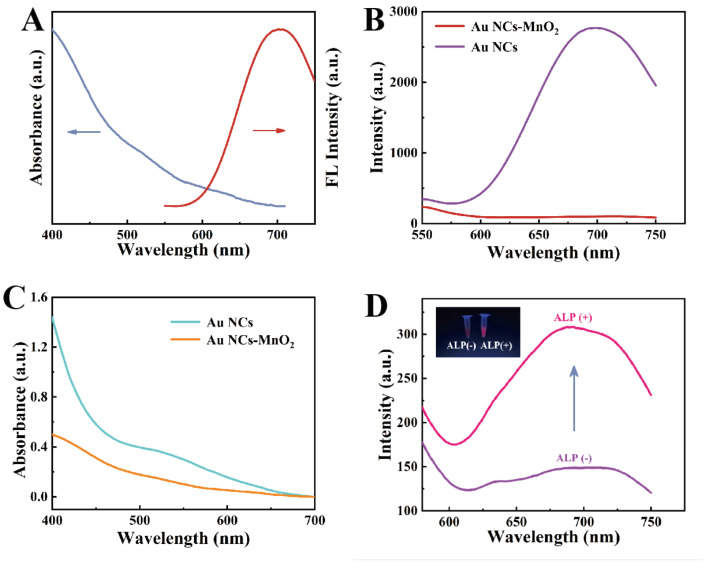
(**A**) UV-vis absorption spectrum of the MnO_2_ NSs (blue line) and the fluorescence emission spectrum of Au NCs (red line); (**B**) the fluorescence emission spectrum of Au NCs and Au NCs-MnO_2_ NSs; (**C**) UV-vis absorption of the spectrum of Au NCs and Au NCs-MnO_2_ NSs; (**D**) Fluorescence spectra of Au NCs-MnO_2_ NSs in the presence and absence of ALP (inset of (**D**) is a photograph of the Au NCs-MnO_2_ NSs in the presence and absence of ALP under UV light).

The feasibility of this assay for detecting ALP were initially studied. As depicted in Figure 3A, Au NCs exhibited distinct fluorescence emission peaks upon excitation with 520 nm, with the maximum emission peak observed at approximately 700 nm. The UV-vis absorption spectrum of MnO_2_ encompassed a broad range that essentially overlapped with the emission spectrum of Au NCs, thereby leading to effective fluorescence quenching of Au NCs through FRET effect. Fluorescence spectra were subsequently employed to validate the quenching capability of MnO_2_ on Au NCs. Results demonstrated that the addition of MnO_2_ NSs reduced the fluorescence intensity of Au NCs from above 2700 to below 200 (Figure 3B and Figure S2). Furthermore, Figure 3C illustrated a significant decrease in absorbance of Au NCs as well. Collectively, these findings unequivocally indicate that MnO_2_ NSs exert a pronounced fluorescence quenching effect on Au NCs.

However, upon the addition of ALP and AAP, the hydrolysis of AAP by ALP generated AA, which in turn reduces MnO_2_ NSs to Mn^2+^ and consequently recorvered the fluorescence of Au NCs. As depicted in Figure 3D, the introduction of ALP significantly enhances the fluorescence emission intensity of Au NCs. The inset clearly demonstrates a pronounced fluorescence emitted from the solution upon addition of ALP under UV light. In conclusion, the assay provides considerable potential for detecting ALP.

### 3.3. Optimization of Experimental Conditions

**Figure 4:**
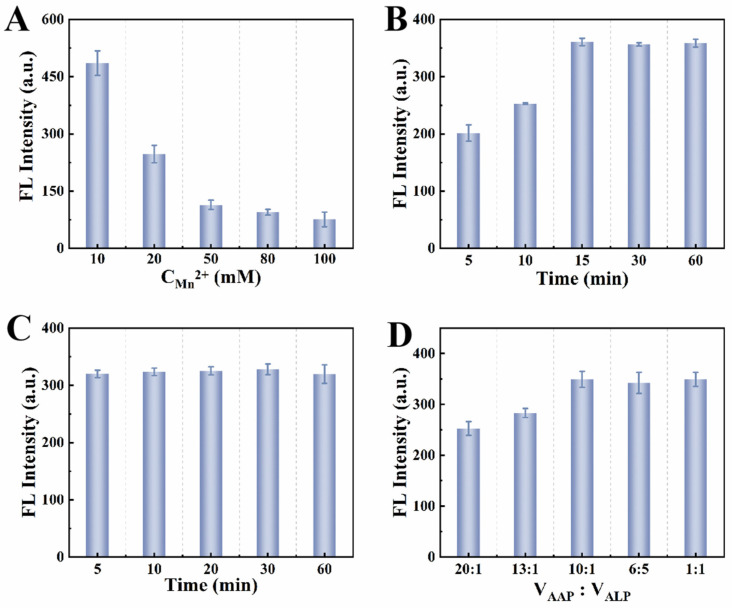
Effect of some experiment conditions on the fluorescence intensity of Au NCs-MnO_2_ NSs system: (**A**) The effect of different concentrations of Mn^2+^ on the synthesis of Au NCs-MnO_2_ NSs; (**B**) incubation time of AAP with ALP; (**C**) reaction time of Au NCs-MnO_2_ NSs with AA; (**D**) different volume ratios of AAP and ALP.

To achieve optimal sensing performance, various experimental conditions were optimized, including the content of MnO_2_ NSs, incubation time of AAP with ALP, reaction time of Au NCs-MnO_2_ NSs with AA, and volume ratio of AAP/ALP. The content of MnO_2_ NSs significantly influences the fluorescence intensity of Au NCs. The concentration of MnSO_4_ was varied in the synthesis process to control the content of MnO_2_ NSs in Au NCs-MnO_2_ NSs. Specifically, concentrations ranging from 10 mM to 100 mM were tested and their impact on fluorescence quenching effect was evaluated. As depicted in Figure 4A, an increase in MnSO_4_ concentration led to a gradual decrease in fluorescence emission intensity of Au NCs, indicating an enhanced quenching effect by MnO_2_ NSs. However, when the concentration reached 50 mM, no significant change in fluorescence emission intensity was observed for Au NCs. Therefore, an optimal concentration of 50 mM for MnSO_4_ was selected. Next, the optimum incubation time of ALP with AAP was investigated. The changes in fluorescence intensity were examined at incubation times of 10, 15, 20, and 30 min. The fluorescence intensity exhibited a gradual recovery with increasing incubation time. After 15 min of incubation, the fluorescence intensity was basically constant (Figure 4B). Based on this optimized incubation time of ALP with AAP, the reaction time for AA reducing MnO_2_ NSs was also studied. As illustrated in Figure 4C, significant recovery of fluorescence intensity was observed after only 5 min of reaction. Prolonging the reaction time did not result in any substantial change in fluorescence intensity. This observation can be attributed to an abundant production of AA through ALP-catalyzed AAP reactions during previous steps, thus enabling rapid reduction of MnO_2_ to Mn^2+^. Finally, concentrations of ALP and AAP were maintained constant while investigating the volume ratio of AAP to ALP. As shown in the Figure 4D, a maximum fluorescence intensity of Au NCs was observed when V_AAP_:V_ALP_ decreases from 20:1 to 10:1. As a result, a volume ratio of 10:1 was determined as the optimal volume ratio for the reaction between AAP and ALP in the following experiments.

### 3.4. Analytical Performance for Detection of ALP

**Figure 5:**
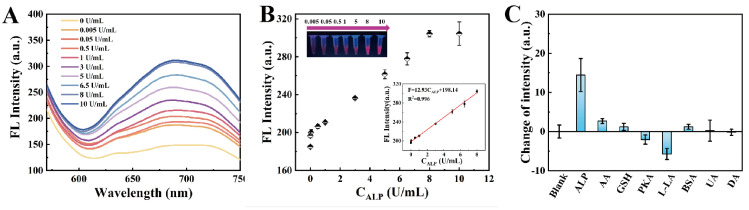
(**A**) Fluorescence signal changes at different concentrations of ALP (0–10 U/mL); (**B**) Fluorescence intensity versus ALP concentration linearly plot (Inset: Images of Au NCs-MnO_2_ NSs toward to various concentrations of ALP under UV); (**C**) Selectivity performance of the assay (n = 3).

The fluorescence signals of Au NCs-MnO_2_ NSs to various ALP concentrations were recorded under optimal detection experiment conditions. As shown in Figure 5A, the fluorescence emission intensity of Au NCs-MnO_2_ NSs gradually increases with the increase of ALP concentration and keep constant when ALP concentration was increased to 8 U/mL. Furthermore, when ALP concentration ranged between 0.005 U/mL and 8 U/mL, the fluorescence emission intensity was proportional to ALP concentration (Figure 5B). The linear regression equation is *F* = 12.93*C_ALP_*(U/mL) + 198.14 (*R*^2^ = 0.996), Where *F* and *C_ALP_* represents the fluorescence emission intensity and the ALP concentration, respectively. The detection limit (LOD) was calculated as 0.0015 U/mL according to *3σ/k*, where *σ* represents the standard deviation of the blank sample and *k* represents the slope of the analytical calibration curve (n = 3). Furthermore, under ultraviolet light, as the concentration of ALP increased from 0.005 U/mL to 8 U/mL, the solution gradually turned into a brighter magenta color. Upon increasing the ALP concentration to 10 U/mL, the solution still exhibited a robust fluorescence (Figure 5B, insert). This further proves that the pronounced recovery of fluorescence is attributed to the increased ALP concentration. In addition, the performance of the assay was compared with other ALP detection methods, as presented in Table S1. The fluorescence assay developed in this study exhibits a remarkably low detection limit and an extended linear range. Moreover, the LOD achieved in this experiment was as low as 5 U/L, significantly lower than the concentration of ALP found in human serum (40–190 U/L), thereby highlighting the potential clinical application of this assay.

### 3.5. Selectivity Performance of Au NCs-MnO_2_ NSs

Good selectivity is a crucial parameter for assessing the performance and potential application of this assay. Thus, AA, DA, UA, GSH, L-LA, PKA, and BSA were chosen as potential interfering substances to validate the specificity of the assay towards ALP detection (Figure 5C). Among them, the first five are reducing substances presence in human serum. The concentrations of AA, DA, and UA were 1 mM, and the concentration of GSH and L-LA were 5 mg/mL and 50 mg/mL, respectively, which were higher than the levels in human serum. PKA was introduced to verify the specificity of ALP catalyzing the dephosphorylation of AAP to produce AA. BSA was utilized as template during the synthesis of Au NCs-MnO_2_ NSs, so BSA (50 mg/mL) was selected for the selectivity experiment. The results demonstrate that the impact of other interfering substances on the fluorescence intensity is negligible in comparison to ALP. Hence, Au NCs-MnO_2_ exhibits good specificity as a fluorescence probe for ALP detection.

### 3.6. Determination ALP in Human Serum

To study the potential application of this assay for the detection of ALP in serum samples, the recovery detection of ALP in human serum was carried out using the standard spiking method. Under the experimental conditions described above, different concentrations of ALP (0.5, 1, 5 U/mL) were added to human serum, and incubated with AAP. According to the linear regression equation, the ALP concentration was calculated and compared with that of the ALP added. The experimental results are presented in Table 1, demonstrating a recovery rate ranging from 93.70% to 105.26% and a relative standard deviation (RSD) within 5%. These results indicated the capability of this assay for detecting ALP in serum and its potential clinical applications.

## 4. Conclusions

In this study, a facile one-pot method was employed to synthesize Au NCs-MnO_2_ NSs using BSA as template, and a “switch-on” fluorescence assay was developed for sensitive detection of ALP. Specifically, Au NCs acted as the fluorescence donor while MnO_2_ NSs served as the acceptor through FRET-mediated quenching of Au NCs. Upon addition of ALP, ALP hydrolyzed AAP to generate AA, leading to effective reduction of MnO_2_ NSs into Mn^2+^ and subsequent fluorescence recovery. This assay exhibited wide detection range (0.005–8 U/mL), high sensitivity (LOD = 0.0015 U/mL), and good selectivity. Moreover, the capability of the assay for detecting ALP in human serum samples was demonstrated, thus holding great potential for clinical applications. Notably, considering the facile detection protocol of this assay, there is a possibility that it can be utilized for point-of-care testing in the future.

## Figures and Tables

**Table 1:** Determination of ALP in human serum samples (n = 3).

Sample	Spiked (U/mL)	Found (U/mL)	Recovery (%)	RSD (%)
1	0.5	0.47	93.70	1.06
2	1	1.05	105.26	2.93
3	5	5.05	101.43	4.23

## Data Availability

The data presented in this study are either included in the manuscript or in the Supporting Information.

## Supplementary Materials

The following supporting information can be downloaded at: https://www.mdpi.com/article/10.3390/bios15010049/s1, Figure S1: HRTEM image of Au NCs; Figure S2: Fluorescence intensity of six different batches of Au NCs quenched by MnO_2_ NSs; Table S1: Comparison analysis of detection performance of ALP using various fluorescence method [31,32,33,34,35,36,37].

## References

[1] Yari A., Penhani S. (2023). A new highly sensitive optical sensor based on Congo-Red for the determination of thorium (IV) in aqueous solution. J. Anal. Test..

[2] Lin C.X., Song X.H., Ye W.L., Liu T., Rong M.C., Niu L. (2024). Recent progress in optical sensors based on MXenes quantum dots and MXenes nanosheets. J. Anal. Test..

[3] Liu Y., Xue R., Yan B. (2025). Development and prospects of covalent organic framework-based ratiometric fluorescent sensors. Coord. Chem. Rev..

[4] Lin Z., Yu W., Hu R., Hu R., Wei Z., Zhang M. (2023). Dual-emission carbonized polymer dots combined with metal ions as a single-component fluorescence sensor array for pattern recognition of glycosaminoglycans. J. Anal. Test..

[5] Zhu R., Du Z., Zhu M., Liang H., Wang S., Zhou Q., Li R., Li Y., Zeng C., Liu W. (2023). Molecularly imprinted polymers embedded with double perovskite quantum dots: A ratiometric fluorescence sensor for visible and fluorescent determination of Rhein. Chem. Eng. J..

[6] Zhang X.F., Wang W.X., Guan L., Chen H., Li D., Zhang L., Huang S.P. (2024). Preparation of a novel green fluorescent carbon quantum dots and application in Fe^3+^ specific detection in biological system. J. Anal. Test..

[7] Yang J., Liu H., Huang Y., Li L., Liu H.J., Ding Y.P. (2024). Carbon dots as “On–Off–On” fluorescence sensors for selective and consecutive detection of 4-nitrophenol and cerium(IV) in water samples. J. Anal. Test..

[8] Wei D., Zhang H., Tao Y., Wang K., Wang Y., Deng C., Xu R., Zhu N., Lu Y., Zeng K. (2024). Dual-emission single sensing element-assembled fluorescent sensor arrays for the rapid discrimination of multiple surfactants in environments. Anal. Chem..

[9] Rong M., Huang Y., Zhuang X., Ma Y., Xie H., Wu Y., Niu L. (2023). And logic-gate-based Au@MnO_2_ sensing platform for tetracyclines with fluorescent and colorimetric dual-signal readouts. Sens. Actuators B Chem..

[10] Chen S., Chen F., Li Y., Wang Y., Wang X., Ye C. (2023). A fluorescein derivative chemosensor combined with triplet–triplet annihilation upconversion system for ratiometric sensing of cysteine. J. Anal. Test..

[11] Zhai T., Zhang Y., Guan D., Yang L., Zhang W., Zhang Y., Liu Q. (2023). Investigation of upconversion luminescence attenuation in aqueous solutions under 980 nm and 808 nm irradiation. J. Anal. Test..

[12] Zhang Q., Zhang Z., Xu S., Liu A., Da L., Lin D., Jiang C. (2023). Photoinduced electron transfer-triggered g-C_3_N_4_\rhodamine b sensing system for the ratiometric fluorescence quantitation of carbendazim. Anal. Chem..

[13] Yan X., Wang Y., Kou Q., Sun Q., Tang J., Yang L., Chen X., Xu W., Le T. (2022). A novel aptasensor based on Fe_3_O_4_/Au/g-C_3_N_4_ for sensitive detection of sulfameter in food matrices. Sens. Actuators B Chem..

[14] Chakraborty S., Mukherjee S. (2021). Effects of protecting groups on luminescent metal nanoclusters: Spectroscopic signatures and applications. Chem. Commun..

[15] Ni S., Liu Y., Tong S., Li S., Song X. (2023). Emerging NIR-II luminescent gold nanoclusters for in vivo bioimaging. J. Anal. Test..

[16] Kim S., Lee E.S., Cha B.S., Park K.S. (2022). High fructose concentration increases the fluorescence stability of DNA-templated copper nanoclusters by several thousand times. Nano Lett..

[17] Hu Y., He Y., Han Y., Ge Y., Song G., Zhou J. (2019). Determination of the activity of alkaline phosphatase based on aggregation-induced quenching of the fluorescence of copper nanoclusters. Microchim. Acta.

[18] Neema P.M., Tomy A.M., Cyriac J. (2020). Chemical sensor platforms based on fluorescence resonance energy transfer (FRET) and 2d materials. TrAC Trend Anal. Chem..

[19] Yang W.C., Li S.Y., Ni S., Liu G. (2024). Advances in FRET-based biosensors from donor-acceptor design to applications. Aggregate.

[20] Sivakumar G., Gupta A., Babu A., Sasmal P.K., Maji S. (2024). Nitrodopamine modified MnO_2_ NS-MoS_2_ QDs hybrid nanocomposite for the extracellular and intracellular detection of glutathione. J. Mater. Chem. B.

[21] Xie M., Gao R., Li K., Kuang S., Wang X., Wen X., Lin X., Wan Y., Han C. (2023). O_2_-generating fluorescent carbon dot-decorated MnO_2_ nanosheets for “off/on” MR/fluorescence imaging and enhanced photodynamic therapy. ACS Appl. Mater. Interfaces.

[22] Xue H., Yu M., He K., Liu Y., Cao Y., Shui Y., Li J., Farooq M., Wang L. (2020). A novel colorimetric and fluorometric probe for biothiols based on MnO_2_ NFs-Rhodamine b system. Anal. Chim. Acta.

[23] Du Y., Liu H., Liang J., Zheng D., Li J., Lan S., Wu M., Zheng A., Liu X. (2020). Protein-assisted formation of gold clusters-MnO_2_ nanocomposite for fluorescence imaging of intracellular glutathione. Talanta.

[24] Ma H., Liu X., Wang X., Li X., Yang C., Iqbal A., Liu W., Li J., Qin W. (2017). Sensitive fluorescent light-up probe for enzymatic determination of glucose using carbon dots modified with MnO_2_ nanosheets. Microchim. Acta.

[25] Cao Y., Song Y., Wei T., Feng T., Li M., Xue C., Xu J. (2024). MnO_2_ in-situ coated upconversion nanosystem for turn-on fluorescence detection of hypoxanthine in aquatic products. Food Chem..

[26] Li Y., Zhang L., Zhang Z., Liu Y., Chen J., Liu J., Du P., Guo H., Lu X. (2021). MnO_2_ nanospheres assisted by cysteine combined with MnO_2_ nanosheets as a fluorescence resonance energy transfer system for “switch-on” detection of glutathione. Anal. Chem..

[27] Mu X., Liu X., Ye X., Zhang W., Li L., Ma P., Song D. (2022). Branched poly(ethylenimine) carbon dots-MnO_2_ nanosheets based fluorescent sensory system for sensing of malachite green in fish samples. Food Chem..

[28] Liu F., Li Z., Kang G., Liu Z., Zhu S., He R., Zhang C., Chen C., Lu Y. (2023). Ratiometric sensing of α-glucosidase and its inhibitor based on MnO_2_ nanosheets promoted in-situ fluorescent reactions. Microchem. J..

[29] Sheng J., Jiang X., Wang L., Yang M., Liu Y.N. (2018). Biomimetic mineralization guided one-pot preparation of gold clusters anchored two-dimensional MnO_2_ nanosheets for fluorometric/magnetic bimodal sensing. Anal. Chem..

[30] Meng H.M., Jin Z., Lv Y., Yang C., Zhang X.B., Tan W., Yu R.Q. (2014). Activatable two-photon fluorescence nanoprobe for bioimaging of glutathione in living cells and tissues. Anal. Chem..

[31] Ma J.L., Yin B.C., Wu X., Ye B.C. (2016). Copper-mediated DNA-scaffolded silver nanocluster on-off switch for detection of pyrophosphate and alkaline phosphatase. Anal. Chem..

[32] Yang J., Zheng L., Wang Y., Li W., Zhang J., Gu J., Fu Y. (2016). Guanine-rich DNA-based peroxidase mimetics for colorimetric assays of alkaline phosphatase. Biosens. Bioelectron..

[33] Qian Z., Chai L., Tang C., Huang Y., Chen J., Feng H. (2015). Carbon quantum dots-based recyclable real-time fluorescence assay for alkaline phosphatase with adenosine triphosphate as substrate. Anal. Chem..

[34] Deng J., Yu P., Wang Y., Mao L. (2015). Real-time ratiometric fluorescent assay for alkaline phosphatase activity with stimulus responsive infinite coordination polymer nanoparticles. Anal. Chem..

[35] Ren X., Chen Z., Chen X., Liu J., Tang F. (2014). Sensitive optical detection of alkaline phosphatase activity with quantum dots. J. Lumines..

[36] Shi W., Li T., Chu N., Liu X., He M., Bui B., Chen M., Chen W. (2021). Nano-octahedral bimetallic Fe/Eu-MOF preparation and dual model sensing of serum alkaline phosphatase (ALP) based on its peroxidase-like property and fluorescence. Mater. Sci. Eng. C.

[37] Hu S., Liu J., Wang Y., Liang Z., Hu B., Xie J., Wong W., Wong K., Qiu B., Peng W. (2023). A new fluorescent biosensor based on inner filter effect and competitive coordination with the europium ion of non-luminescent eu-mof nanosheets for the determination of alkaline phosphatase activity in human serum. Sens. Actuators B Chem..

