# Supplementary material for: BSA-Assisted Synthesis of Au Nanoclusters/MnO2 Nanosheets for Fluorescence “Switch-On” Detection of Alkaline Phosphatase
Source: Biosensors (Basel). 2025 Jan 15;15(1):49. doi: 10.3390/bios15010049 (PMC11763645; doi:10.3390/bios15010049)
Supplement: Supplementary file 1 [file biosensors-15-00049-s001.zip › biosensors-3391558-supplementary.pdf]

Supporting information for

BSA-assisted synthesis of Au nanoclusters/MnO_2 nanosheets based fluorescence probe for "switch-on" detection of alkaline phosphatase

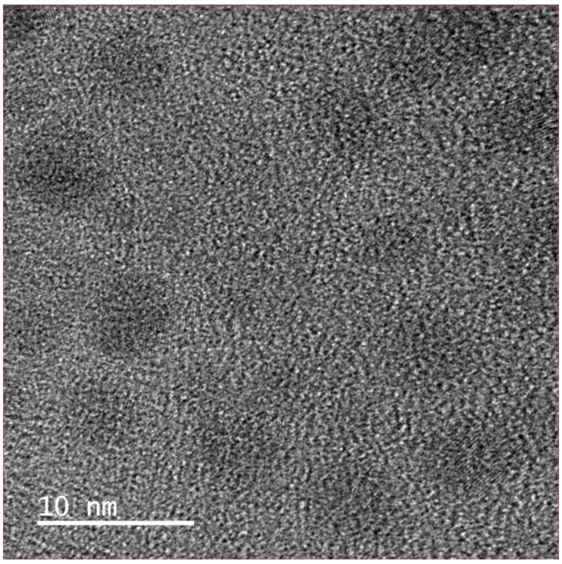

Figure S1. HRTEM image of Au NCs

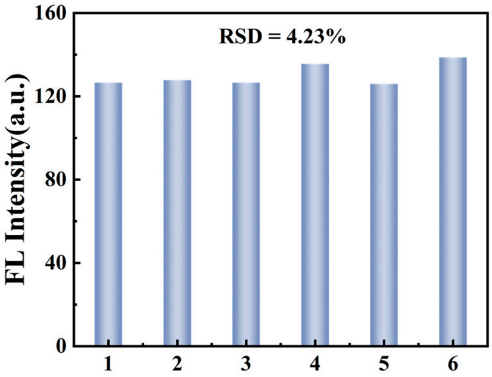

Figure S2. Fluorescence intensity of six different batches of Au NCs quenched by MnO_2 NSs

Table S1. Comparison of the detection performance for the fluorescence detection of ALP.

Matrials	Linear range (U/L)	LOD (U/L)	Ref.
Silver Nanoclusters	30-240	5	[1]
G_{20}-Cu(II) complex	20-200	0.84	[2]
carbon quantum dots (CQDs)	4.6-383.3	1.4	[3]
Infinite Coordination Polymer (ICP) NPs	25-200	10	[4]
CdTe/CdS QDs	3-1000	3	[5]
Fe/Eu-MOF	1-200	0.9	[6]
nonluminescent lanthanide-metal organic framework nanosheets (NO_2-Eu-MOF NS)	5-1000	1.1	[7]
Au NCs/MnO_2 NSs	5-8000	1.5	This work

Reference

1. Ma, J.L.; Yin, B.C.; Wu, X.; Ye, B.C. Copper-mediated dna-scaffolded silver nanocluster on-off switch for detection of pyrophosphate and alkaline phosphatase. *Anal. Chem.* **2016**, *88*, 9219-9225.
2. Yang, J.; Zheng, L.; Wang, Y.; Li, W.; Zhang, J.; Gu, J.; Fu, Y. Guanine-rich dna-based peroxidase mimetics for colorimetric assays of alkaline phosphatase. *Biosens. Bioelectron.* **2016**, *77*, 549-556.
3. Qian, Z.; Chai, L.; Tang, C.; Huang, Y.; Chen, J.; Feng, H. Carbon quantum dots-based recyclable real-time fluorescence assay for alkaline phosphatase with adenosine triphosphate as substrate. *Anal. Chem.* **2015**, *87*, 2966-2973.
4. Deng, J.; Yu, P.; Wang, Y.; Mao, L. Real-time ratiometric fluorescent assay for alkaline phosphatase activity with stimulus responsive infinite coordination polymer nanoparticles. *Anal. Chem.* **2015**, *87*, 3080-3086.
5. Ren, X.; Chen, Z.; Chen, X.; Liu, J.; Tang, F. Sensitive optical detection of alkaline phosphatase activity with quantum dots. *J. Lumines.* **2014**, *145*, 330-334.
6. Shi, W.; Li, T.; Chu, N.; Liu, X.; He, M.; Bui, B.; Chen, M.; Chen, W. Nano-octahedral bimetallic fe/eu-mof preparation and dual model sensing of serum alkaline phosphatase (alp) based on its peroxidase-like property and fluorescence. *Materials Science and Engineering: C* **2021**, *129*, 112404.
7. Hu, S.; Liu, J.; Wang, Y.; Liang, Z.; Hu, B.; Xie, J.; Wong, W.; Wong, K.; Qiu, B.; Peng, W. A new fluorescent biosensor based on inner filter effect and competitive coordination with the europium ion of non-luminescent eu-mof nanosheets for the determination of alkaline phosphatase activity in human serum. *Sensors and Actuators B: Chemical* **2023**, *380*, 133379.
